# Facilitation of Balsam Fir by Trembling Aspen in the Boreal Forest: Do Ectomycorrhizal Communities Matter?

**DOI:** 10.3389/fpls.2019.00932

**Published:** 2019-07-18

**Authors:** Mélissande Nagati, Mélanie Roy, Annie Desrochers, Sophie Manzi, Yves Bergeron, Monique Gardes

**Affiliations:** ^1^UQAT-UQAM Industrial Chair in Sustainable Forest Management, Forest Research Institute, University of Québec in Abitibi-Témiscamingue, Rouyn-Noranda, QC, Canada; ^2^UMR5174, Laboratory Evolution and Biological Diversity, Centre National de la Recherche Scientifique – IRD, Université Paul Sabatier, Toulouse, France

**Keywords:** *Abies balsamea*, boreal forest, ectomycorrhiza, ericaceous shrubs, facilitation, *Picea mariana*, *Populus tremuloide*s

## Abstract

Succession is generally well described above-ground in the boreal forest, and several studies have demonstrated the role of interspecific facilitation in tree species establishment. However, the role of mycorrhizal communities for tree establishment and interspecific facilitation, has been little explored. At the ecotone between the mixed boreal forest, dominated by balsam fir and hardwood species, and the boreal forest, dominated by black spruce, several stands of trembling aspen can be found, surrounded by black spruce forest. Regeneration of balsam fir seems to have increased in the recent decades within the boreal forest, and it seems better adapted to grow in trembling aspen stands than in black spruce stands, even when located in similar abiotic conditions. As black spruce stands are also covered by ericaceous shrubs, we investigated if differences in soil fungal communities and ericaceous shrubs abundance could explain the differences observed in balsam fir growth and nutrition. We conducted a study centered on individual saplings to link growth and foliar nutrient concentrations to local vegetation cover, mycorrhization rate, and mycorrhizal communities associated with balsam fir roots. We found that foliar nutrient concentrations and ramification indices (colonization by mycorrhiza per length of root) were greater in trembling aspen stands and were positively correlated to apical and lateral growth of balsam fir saplings. In black spruce stands, the presence of ericaceous shrubs near balsam fir saplings affected ectomycorrhizal communities associated with tree roots which in turn negatively correlated with N foliar concentrations. Our results reveal that fungal communities observed under aspen are drivers of balsam fir early growth and nutrition in boreal forest stands and may facilitate ecotone migration in a context of climate change.

## Introduction

Tree establishment, growth and survival in a new area are primarily dependent upon seed availability and local environmental conditions. For a given tree species, its capacity to establish under new biotic conditions is predominantly driven by either facilitation or competitive processes (Callaway and Walker, 1997). Within a multi-species environment, species can compete for resources such as light, nutrients or water. At the same time, survival of one species can be facilitated by another species providing protection against predators or extreme climate events (Stachowicz, 2001), or by the presence of their mycorrhizal symbionts which could result in mycorrhizal networks (Simard et al., 2015; Pickles and Simard, 2017), or exchanges of resources (Brooker et al., 2008; Teste et al., 2009). Among the consequences of climate change, changes in species distribution are already observed and could result in new or modified species interactions. Changes in distribution range of species are particularly visible at ecotones between forested ecosystems (Messaoud et al., 2007; Barbeta and Peñuelas, 2017) or at the northern tree line (Harsch et al., 2009; Ratcliffe et al., 2017).

In Québec (Canada), the 49th parallel represents the ecotone between the southern balsam fir-paper birch (mixedwood boreal forest) and the northern black spruce-feather moss (boreal forest) bioclimatic domains. It has been suggested that this ecotone is likely to migrate northward with climate change (Messaoud et al., 2007). South of this ecotone, forest stands are mixed and dominated by *Abies balsamea* [L.] Mill. (balsam fir-BF), *Picea glauca* [Moench] Voss (white spruce), *Populus tremuloides* Michx.(trembling aspen-TA), and *Betula papyrifera* Marsh. (paper birch), whereas *Picea mariana* [Mill.] BSP (black spruce-BS), and *Pinus banksiana* Lamb.(Jack pine) dominate north of the ecotone (Robitaille and Saucier, 1996). In the southern part of the black spruce-feather moss domain, small stands of trembling aspen occur, in which trembling aspen can be dominant (more than 75% of the canopy cover) or mixed with black spruce (between 25 and 75% of the canopy cover; Légaré et al., 2005; Cavard et al., 2011). Following classical forest dynamics in these forests, TA would eventually be replaced by BS in the absence of disturbance (Lecomte and Bergeron, 2005; Belleau et al., 2011), thereby maintaining BS dominance in the area. In areas where balsam fir establishes under BS- and TA-dominated stands, a greater abundance of more vigorous saplings has been observed under TA (Arbour and Bergeron, 2011). As those two types of stands are very close geographically and grow under similar climate, slope and substrates (Légaré et al., 2005; Cavard et al., 2011), the idea of an abiotic determinism could be rejected. On the contrary, aspen-dominated and spruce-dominated stands have different understorey plant and fungal communities, and soil organic contents (Légaré et al., 2005; Cavard et al., 2011; Nagati et al., 2018), suggesting that biotic factors could explain differences in balsam fir growth. Indeed several mechanisms resulting from microbe–mediated interactions could enhance or reduce BF nutrition and growth, and may be involved in plant–plant interactions in the boreal forest.

Among the fungi that interact with tree roots, including BF roots, ectomycorrhizal fungi (EMF) are particularly abundant in boreal soils (Taylor et al., 2013). These fungi improve tree growth and foliar nutrient status in ecosystems where nutrients are mainly found in organic forms, as in boreal forests (Smith and Read, 2008; Inselsbacher and Näsholm, 2012; Franklin et al., 2014). At a worldwide scale, closely related host species tend to share more similar EMF communities than more phylogenetically distant host species (Tedersoo et al., 2013), which may result in greater similarity between BF and BS fungal communities. However, at the local scale, cases of facilitation through mycorrhizal symbioses have rather been detected between phylogenetically distinct plants (Nara and Hogetsu, 2004; Dickie et al., 2006; van der Heijden and Horton, 2009). Mycorrhizal fungi could also shape plant–plant interactions through indirect interactions with plants (Bever et al., 2010). The understorey vegetation under TA is dominated by various ectomycorrhizal tree saplings, endomycorrhizal (AM) shrubs and herbaceous species (Légaré et al., 2005; Cavard et al., 2011) while BS stands understorey is covered by a thick layer of bryophytes and ericaceous shrubs. Limitations to tree establishment and growth for EMF tree species that are adjacent to ericaceous shrubs, which are associated with ericoid fungi, have been documented many times in various ecosystems (e.g., Peterson, 1965; Zackrisson et al., 1997; Yamasaki et al., 1998; Walker et al., 1999; Mallik, 2003). Although the mechanisms explaining such regeneration failures of EMF plant species near ericoid shrubs are not clearly understood (Peterson, 1965; Gallet, 1994; Collier and Bidartondo, 2009; Richard et al., 2009), potential alterations to BF mycorrhizal colonization and mycorrhizal networks cannot be excluded in our system that would explain BF growth differences between TA- and BS-dominated stands.

Our aim was to disentangle the processes controlling BF early growth and nutrition at this boreal ecotone. To achieve this goal, we monitored growth parameters of individual BF saplings for two consecutive years and investigated their EMF communities and their foliar nutrient concentrations under BS and TA stands. We hypothesized that (1) local conditions under TA would lead to greater mycorrhization rates and different EMF communities than under BS and (2) the presence of ericaceous shrubs near BF saplings would reduce growth and mineral nutrition through their effects on EMF symbioses.

## Materials and Methods

### Site Description and Fir Sapling Selection

The study was located across a 36-km^2^ area within the Clay Belt of northern Québec and Ontario (Canada). Four paired and unmanaged sites within the black spruce-feather moss domain were selected for study (Table 1). Each pair of stands contained one that was dominated by TA, while the other one was dominated by BS (for more information on sites, see Nagati et al., 2018). At each site, 15 BF saplings that were 25–75 cm tall and separated from one another by at least 5 m were selected: five in TA stands; five in BS stands at least 4m away from an ericaceous shrub; and five in BS stands at a maximum distance of 3 m from an ericaceous shrub [BSE, Labrador tea (*Rhododendron groenlandicum* [Oeder] Kron and Judd) or sheep-laurel (*Kalmia angustifolia* L.)]. Our sampling design permitted to sample 15 saplings per site (10 in BS stands and 5 in TA stands), resulting in 60 saplings.

**TABLE 1:** GPS coordinates of sites.

**Site**	**Stand**	**Latitude**	**Longitude**
1	BS	49.19061	−78.82797
	TA	49.18942	−78.82817
2	BS	49.19367	−78.83556
	TA	49.19375	−78.8345
3	BS	49.196695	−78.842092
	TA	49.196422	−78.84237
4	BS	49.168972	−78.885194
	TA	49.180417	−78.883611

*BS, black spruce – *Picea Mariana*; TA, trembling aspen – *Populus Tremuloides*.*

### Balsam Fir Measurements

Annual apical growth (cm) was measured in August 2015 and 2016 with calipers and the two measures were summed. Annual lateral growth was measured on two randomly selected branches per sapling in August 2015 and 2016 with calipers, averaged by year, and then summed. In 2016, the basal diameter of stems (cm) was measured with calipers. Fir needles were harvested in August 2016, forced air-dried at 30°C for 12 h, and sent to the Laurentian Forestry Centre, Canadian Forest Service (Quebec) for chemical analyses. Percentage foliar C and N were measured with a Leco TruMac CNS mass spectrometer (LECO Corporation, St. Joseph, MI, United States). Minor and major cation concentrations (g/kg of needles) were obtained by ICP-OES with a Perkin-Elmer Optima 7300 DV (Waltham, MA, United States), following the method proposed by Kalra (1998).

### Characterization of Local Biotic Environment

Given that the main goal of our study was to determine whether biotic interactions affected BF growth and foliar nutritional status, we characterized their local biotic environment. Forest composition within a 3 m radius around each balsam fir sapling was evaluated by measuring the percentages of TA, BS and BF relative to their diameter at DBH (diameter at breast height, 1.3 m). Only trees >5 cm DBH were taken into account. These data allowed to calculate the percent of each tree species (TA, BS, and BF) in the canopy around each BF sapling.

### Mycorrhizal Root Tip Counts, Mycorrhizal DNA Extraction, Amplification, and Sequencing

Mycorrhizal communities were assessed for each sapling to test their effect on individual BF growth and foliar nutrient status. The root system of each sapling was gently excavated in August 2016 and washed to preserve mycorrhizal root tips. Mycorrhizal root tip counts were performed the same day that the roots were extracted. For each sapling, we randomly selected three 10-cm root fragments for live mycorrhizal root tip counts under a magnifying glass to calculate mycorrhization rate (number of EMF root tips/total number of root tips) and ramification index (number of EMF root tips per 10 cm root). For each fir sapling, 50 mycorrhizal root tips were sampled and stored in CTAB 2X at −20°C until DNA extraction. Root tips were pooled by tree sapling and manually ground with a pestle before DNA extraction. DNA was extracted with a PowerSoil DNA extraction kit (MoBio, Carlsbad, CA, United States) following the manufacturer’s instructions. A negative blank extraction (extraction without any material) was performed for every set of 23 extractions. In addition, a tool control was performed on tools that were used for field and laboratory work by extracting DNA from distilled water that was used to wash tools. DNA amplification was performed using the method described in Nagati et al. (2018). Briefly, the fungal ITS1 region (Forward: ITS5 GGAAGTAAAAGTCGTAACAAGG, White et al., 1990; and a modified version of Reverse: 5.8S_Fungi CAAGAGATCCGTTGTTGAAAGTK, Epp et al., 2012) was amplified for 35 PCR cycles. PCR samples were sent to GENETOUL GetPlaGe (Toulouse, France) for sequencing on an Illumina MiSeq platform with the TruSeq Nano PCR-free kit. Sequencing was conducted using the paired-end sequencing technology (2 × 250pb) with the chemistry V2.

### Bioinformatics and Sequence Analysis

An abundance matrix was constructed with *OBITool* packages (Boyer et al., 2016) and R script (R Core Team, 2018, version 3.2.3, 2017) based upon the occurrence of sequences among samples. We performed read-pairing assembly, read attribution to samples, read dereplication, and removal of low-quality sequences (shorter than expected, containing ambiguous nucleotides, displaying low score paired-end alignments, or corresponding to singletons). *OBITool Sumaclust* was used to cluster sequences as OTUs (Operational Taxonomic Units) at a 97% identity threshold (Nilsson et al., 2008). Taxonomic assignment was performed with the *OBITool Ecotag* function against the GenBank extracted database^1^. As was the case for our previous dataset that was collected in the same geographic area (Nagati et al., 2018), taxonomic assignments were more accurate with Genbank than with the UNITE public database^2^. Trophic guild assignment was based upon FUNguild software outputs (Nguyen et al., 2016). Sequences belonging to the same OTU were then summed by sample. Lastly, we removed OTUs that were dominant (with the highest read count) in negative or tool extraction and amplification controls, OTUs not belonging to fungi, or OTUs with coarse-resolution taxonomic assignments (i.e., assigned to Eukaryota).

### Statistical Analyses

Statistical analyses were performed in R (R Core Team, 2018). Data are available at Dryad repository (doi: 10.5061/dryad.914j5m0) Our main goal was to compare individual traits and fungal communities among sapling types (BS, BSE, and TA). Abundance of each OTU was used to avoid giving too much importance to rare OTUs, as recommended for fungal ITS (Unterseher et al., 2011; Lindahl et al., 2013). Ectomycorrhizal species richness, Shannon index (*H*’), ramification index and mycorrhization rate were each calculated and compared according to one-way ANOVA (three levels), followed by Tukey *post hoc* tests of the means. Foliar nutrient status among saplings growing under BS, BSE, and TA, were compared for each nutrient (N, P, K, Ca, and Mg) with non-parametric Kruskall–Wallis tests, given that the data were not normally distributed, followed by Dunn’s *post hoc* tests with Bonferroni corrections. As well, sapling growth between the three modalities was compared with Kruskall-Wallis tests and Dunn’s *post hoc* tests with Bonferonni corrections.

Differences in abundance of each ectomycorrhizal family represented in root tip samples were tested between sapling types with Kruskall–Wallis tests, followed by Dunn *post hoc* tests with Bonferroni corrections. We only tested differences in abundance at the family level, given that about 30% of OTUs could not be assigned to a genus. Differences in ectomycorrhizal community structure between the roots of the three sapling types were visually described with Non-Metric Multidimensional Scaling (NMDS, *vegan* package in R; Oksanen et al., 2017) and coordinates of scores on the first two axes of NMDS were extracted for further analyses. Correlation between the NMDS space and individual measures of fir saplings (nutrient concentrations and growth) were tested with envfit (vegan package). Envfit vectors of individual measures were plotted on the NMDS space when *p*-Values where significant (*p* < 0.05). Differences in ectomycorrhizal community structure between sites, dominant plant communities (BS, BSE, and TA) were tested with PERMANOVA (*Adonis* function, *vegan* package; Oksanen et al., 2017) with nested factors (site/plant community).

### Path Analysis

To test direct and indirect effects of the local biotic environment on growth (for this analysis, growth measures from 2015 to 2016 were summed) and foliar N concentrations, we compared three hypotheses by fitting structural equation models (SEM) to the data (with lavaan package; Rosseel et al., 2018). We included only foliar N concentrations in our model, as it was the only nutrient concentration available for all saplings. We constructed three SEMs to represent three *a priori* hypotheses. Each of the hypotheses is rooted in current knowledge regarding the processes that have been described in the literature, and which are described below. All models were fitted to centered and reduced data.

#### Complete Model Mod1

Considering direct links, we formulated five hypotheses. The first was that foliar N concentration was positively correlated with growth (Pallardy and Kozlowski, 2008). The second one was that EMF abundance and communities were positively correlated with growth and foliar N concentrations (Smith and Read, 2008). The third hypothesis was that BS stands and the percent of BS near fir saplings were negatively correlated with BF growth and foliar N concentrations (Arbour and Bergeron, 2011). The fourth hypothesis was that the presence of ericaceous shrubs near saplings was negatively correlated with BF growth and nutrition (Peterson, 1965; Yamasaki et al., 1998; Mallik, 2003). Finally, we hypothesized that the percentage of conspecific mature trees near BF saplings was positively correlated with their growth and foliar N, given that local conditions have permitted their growth and survival. Lateral and apical growth were added as co-variables in the model and together co-varied with basal diameter of BF saplings.

**FIGURE 1:**
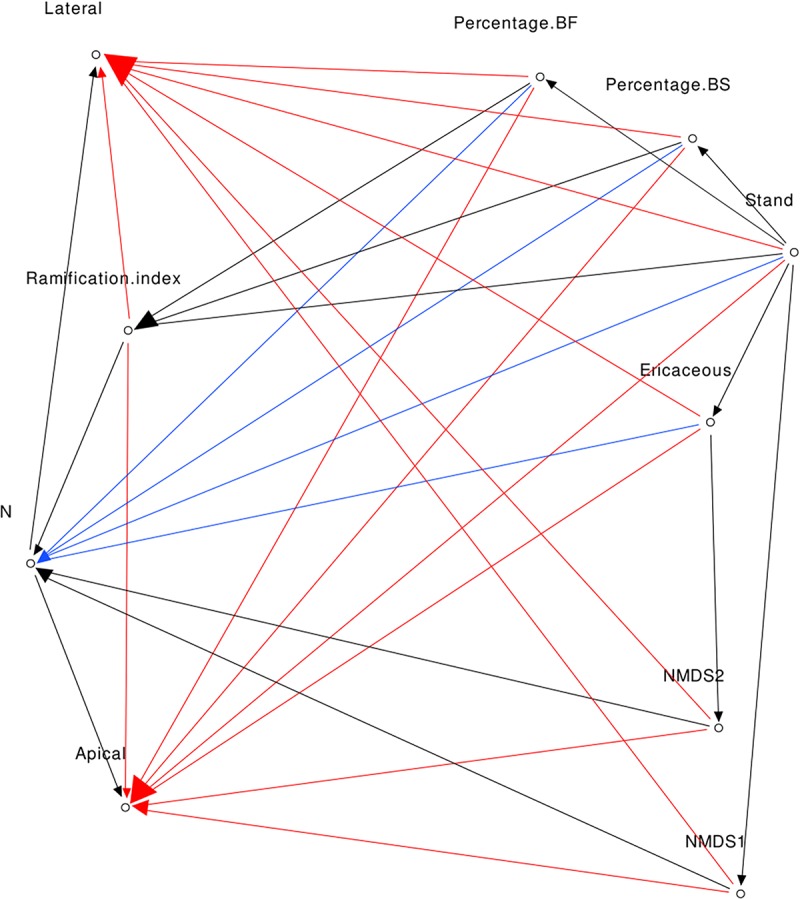
Direct acyclic graph of Mod1 (red, blue, and black arrows), Mod2 (blue and black arrows), and Mod3 (black arrows).

For this model, we also formulated indirect linkage hypotheses. The relative percentage of BF was correlated with stand type (Arbour and Bergeron, 2011). The relative percentage of BS was correlated with the stand (Cavard et al., 2011). The presence of ericaceous shrubs was correlated with the stand type (BF saplings in TA stands were never next to ericaceous shrubs). The ramification index was correlated with the stand (see Figure 3), and percent of BF (the presence of mature trees near BF saplings increased the probability of encountering EMF partners). The NMDS first axis was correlated with the stand and the second axis negatively with the presence of ericaceous shrubs (see Figure 5 and PERMANOVA results). The complete model is presented in Figure 1.

#### Nutrient Model Mod2

The indirect links are the same as for Mod1 but in this model we assumed that foliar N concentration was the only variable that was correlated to growth.

#### Fungi Centered Model Mod3

The links are the same as for Mod2, but in this model we assumed that only variables linked to EMF were correlated with foliar N concentrations. In this model, NMDS first and second axes and ramification index were the only variables directly linked to N concentrations.

To ensure that our models respected independence between non-linked variables, we performed Fisher’s C test (*ggm* package, Marchetti et al., 2015). Models with *p*-Values greater than 0.05 are considered to have respected claims of independence (Shipley, 2000). A model was considered to be representative of the population if the *p*-Value of the Chi-square test was greater than 0.05. For each model, we calculated the Comparative Fit Index (CFI) and the Tucker-Lewis Index (TLI) to evaluate how each model fits to the data. Values greater than 0.95 were considered to be good fits (Hu and Bentler, 1999). Each structural equation model with Fisher’s C test and Chi-Square *p*-values > 0.05, CFI > 0.95, and TLI > 0.95 could describe the data well, we used these values as a primary filter to ensure that models fit well the data and are representative of the population. After applying this filter our aim was to select the best model based on AIC criterion, however, only Mod1 passed the primary filter and was de facto selected.

## Results

### Fir Sapling Traits and Differences Between Stands

Given that 6 of the 60 BF saplings were missing after the first year of fieldwork, the results presented here are based on 18 saplings for each sapling type (i.e., 54 saplings). For 11 saplings, the quantity of needles was insufficient to measure minor and major cations, thereby further reducing the sample size.

**FIGURE 2:**
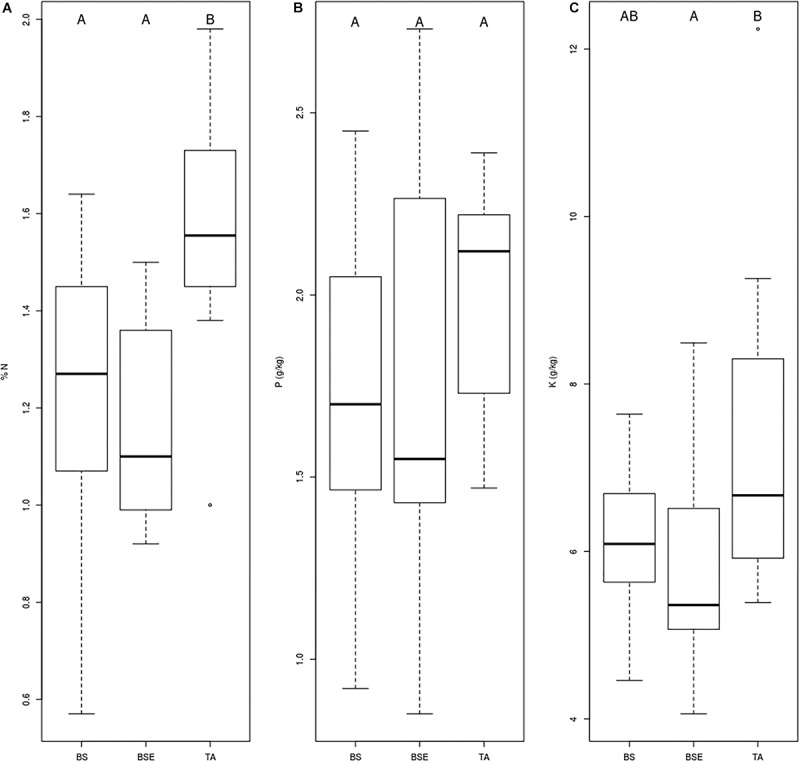
Boxplot chart representing foliar **(A)** N, **(B)** P, and **(C)** K concentrations of BF needles sampled in different stands (BS, black spruce; BSE, black spruce plus ericaceous shrubs; TA, trembling aspen). Different letters indicate differences between modalities.

**FIGURE 3:**
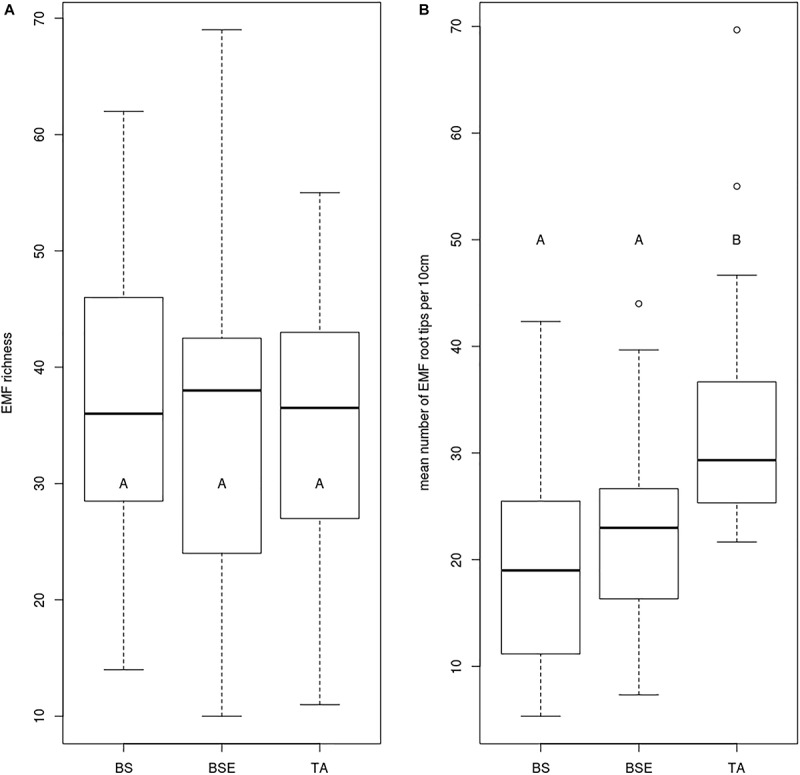
Boxplot charts showing **(A)** EMF richness and **(B)** number of EMF root tips per 10 cm roots of BF saplings sampled in different stands. BS, black spruce; BSE, black spruce plus ericaceous shrubs; TA, trembling aspen. Different letters indicate differences between modalities.

Mean foliar N concentrations of BF saplings were highest in TA stands compared to BS and BSE stands (*p* < 0.05, Figure 2A). Mean foliar K concentration was higher in TA than in BSE stands (*p* < 0.05, Figure 2C). Foliar P (Figure 2B), Ca, and Mg did not vary between sapling types (*p* > 0.05). No significant differences were found in root tip EMF richness and Shannon index among sapling types (Figure 3A, *p* > 0.05). Mean lateral growth of fir saplings did not differ between stands in 2015 and 2016. Apical growth was greater in TA than in BSE stands in 2015, while no difference was detected for apical growth in 2016. Summed lateral and apical growth did not differ between stands. Ramification index of BF roots was higher in TA than in other stands (Figure 3B, *p* < 0.05).

### Ectomycorrhizal Community

**FIGURE 4:**
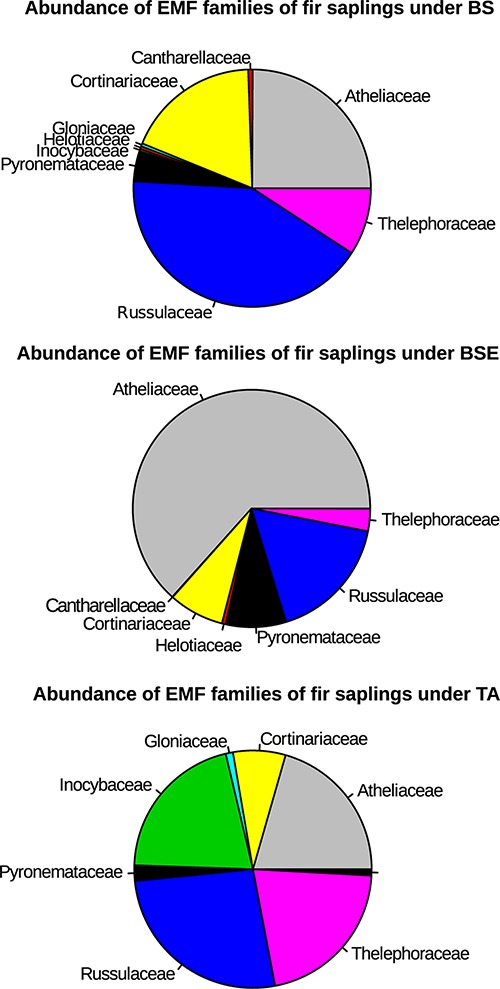
Pie chart representing percent of EMF families per stand type for root tips samples of BF saplings (based on the abundance of reads). BS, black spruce; BSE, black spruce plus ericaceous shrub; TA, trembling aspen.

**FIGURE 5:**
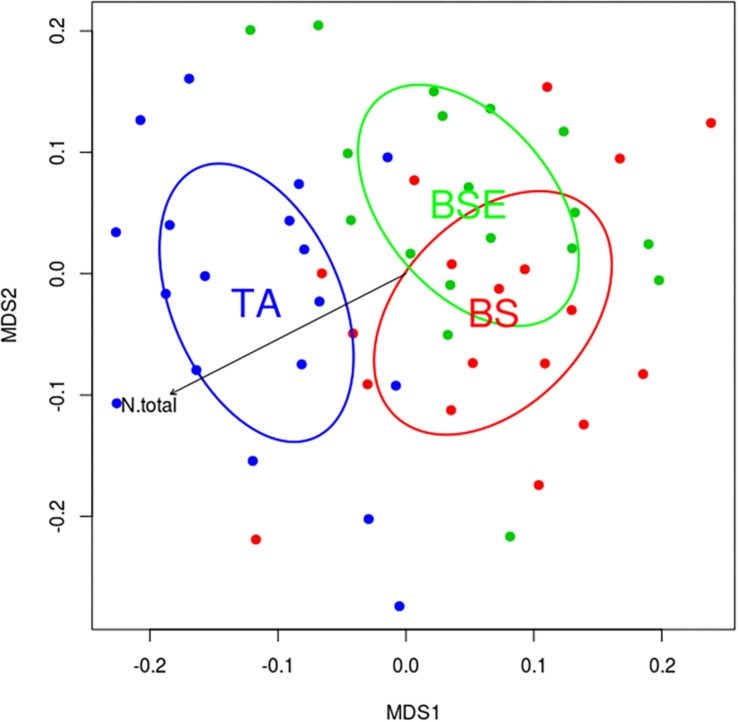
Non-metric multidimensional scaling (NMDS) plots representing similarity between EMF communities of BF root tips in black spruce (BS, red), black spruce + ericaceous shrubs (BSE, green) and trembling aspen (TA, blue) stands. Arrow represents the correlation between N concentration in fir needle and NMDS space.

A total of 400 EMF OTUs (655,495 reads) were found in EMF root tips from BF saplings, representing 19.5% of OTUs and 59.7% of reads, with an average of 35.4 OTUs and 12,056.8 reads per sample. Variation in EMF root tip communities was explained by plant community (*p*-Value = 0.035, *F* = 1.1852, *R*^2^ = 0.169), but no effect of site was detected (Table 2). Clavulinaceae were only found under TA. Further, the abundance of Cortinariaceae was higher under BS compared to TA stands, that of Gloniaceae was higher under TA than under BSE, and that of Helotiaceae was higher under BSE than under TA. Finally, abundance of Inocybaceae and Thelephoraceae were greater under TA than under BS and BSE (Kruskal-Wallis and Dunn *post hoc* tests, *p* < 0.025, Figure 4). According to the NMDS, EMF communities on root tips from the TA stands were distinct from those under BS and BSE, while communities in BS and BSE overlapped (Figure 5). *Envfit* test demonstrated that N concentration were significantly associated with NMDS structure (*p* < 0.05, Figure 5).

**TABLE 2:** Results of PERMANOVA conducted on EMF community of balsam fir saplings.

	***df***	**F-model**	***R*^2^**	***P*-value**
Site	3	1.1749	0.06437	0.104
Site: plant community	8	1.1852	0.16855	0.035
Residuals	42	–	0.76708	–

### Path Analysis

**FIGURE 6:**
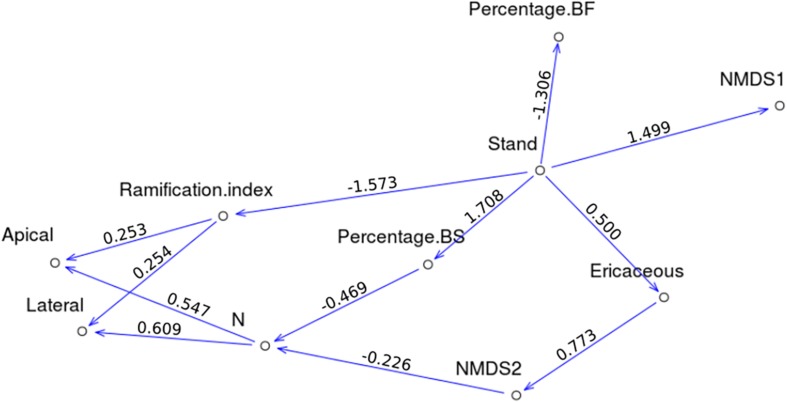
Direct acyclic graphs corresponding to Mod1, only significant links between variables are shown, path coefficients are indicated above each arrow.

The *p*-Values of the Fisher’s *C*-tests were greater than 0.05 for the three models, indicating that all models could be accepted. The *p*-Values of the associated Chi-Square tests ranged between 0.019 and 0.401. CFI ranged between 0.930 and 0.997, while those for TLI ranged between 0.901 and 0.991 (Table 3). Given these results, the interactions model Mod1 was the only one passing the primary filter. Here, we present direct and indirect links for which *p*-Values were significant at *p* < 0.05 (for a summary of the parameter estimates, see Supplementary Table 1). Apical and lateral growth was directly correlated with foliar N concentrations (path coefficients were 0.547 and 0.609, respectively) and the ramification index (0.253 and 0.254). Of interest, foliar N concentrations were negatively correlated with NMDS second axis (−0.226) and the percentage of BS (−0.469). The ramification index was negatively correlated with BS stand (−1.573). NMDS first axis was positively correlated with BS stands (1.499), while the second axis was positively correlated with the presence of ericaceous shrubs (0.773). The presence of ericaceous shrubs, in turn, was positively correlated with BS stands (0.500). Percentage of BS was positively correlated with BS stands (1.708), while the percentage of BF was negatively correlated with BS stands (−1.306). Significant direct and indirect links between variables are presented in Figure 6. All significant and non-significant parameter estimates are presented in Supplementary Table 1.

**TABLE 3:** Statistics of each structural equation models.

**Model**	***P*-value (Chisq)**	**CFI**	**TLI**
Mod1	0.401	0.997	0.991
Mod2	0.103	0.962	0.941
Mod3	0.019	0.930	0.901

## Discussion

The role of biotic interactions was investigated here to explain observed growth differences in balsam fir saplings growing at a northern ecotone of the boreal forest. We hypothesized that biotic interactions could explain the greater nutrition under aspen than under spruce. Our results suggest that facilitation through ectomycorrhizal fungi, together with competition with ericaceous shrubs under BS, are significant drivers of BF growth and nutrition.

As soil EMF communities were strongly divergent between TA and BS-dominated stands (Nagati et al., 2018), we hypothesized that young BF would associate with distinct EMF in the two stands, and that BF would have greater mycorrhization rates under TA stands. Contrary to our hypothesis, observation of BF root tips revealed EM root tips in all conditions, with similar mycorrhization rate. Sequencing of EM root tips confirmed our observation as species richness was not different between stands. We rather detected differences of root architecture and community composition. Indeed, we observed a higher ramification index under TA than under BS stands, probably leading to differences in abilities to explore soil for resources. Our path analyses suggested that soil conditions in TA stands led to a higher ramification index, which in turn improved BF early growth, likely by enhancing foliar nutrient concentrations. Moreover we detected that percent BS around saplings was negatively correlated to sapling N content, this is probably linked to the greater N availability in TA than in BS stands (Nagati et al., 2018). As demonstrated by path analysis, N availability (reflected by the stand and so on, by the percent of BS) was not the only factor affecting sapling needle N content and EMF community structure was also correlated to N nutrition. There are therefore complex interactions leading to a better nutrition of young BF, and path analyses highlighted the importance of both stand dominance and EMF community.

Between stands, communities of EMF on BF roots were not distinct in species richness but rather in composition, and for example Clavulinaceae were only detected under TA stands. Based on soil sequencing, our previous study also revealed a difference of composition but not species richness between stands (Nagati et al., 2018). More generally, changes in plant dominance rather shape EMF community structure than the species richness (Tedersoo et al., 2012). Such a change in EMF community can have strong functional consequences. Based on our results, changes in community composition were correlated with changes in N concentration. We detected a higher abundance of Inocybaceae and Thelephoraceae in BF roots tips in TA than in BSE and BS stands, and Helotiaceae were more abundant in BSE than TA stand. These differences of EMF abundance between stands could partly explain differences in N uptake by BF saplings. Depending on site conditions, different EMF species or assemblage could have different abilities to uptake nutrients in soil and to transfer these nutrients to plants (Jonsson et al., 2001; Buée et al., 2007). This result could suggest that some fungi are more efficient provider of N to young BF. Nara (2006) experiments illustrated how variable can be the result of EM interactions with distinct EMF fungi. By artificially connecting two different tree species through a common mycorrhizal network from different EMF species, Nara (2006) showed that N nutrition could be 6 times higher when trees were connected by *Hebeloma mesophaeum* as compared to trees connected by *Laccaria amethystina*. As BF needles were generally richer in N under TA, the question remain if this benefit under TA could be due to N transfer through a common mycorrhizal network (Simard et al., 2012) involving BF and TA, or only to better soil conditions under TA. We did not sequence TA roots, to detect possible shared fungi and experimental manipulations may be necessary to test whether TA could bring a direct benefit to young BF saplings. As suggested by the models of Bever et al. (2010), the facilitation of BF by TA may not only be explained by shared fungi, but simply by changes in EMF abundance, which we detected between the two stands. The higher abundance of Helotiaceae associated with BF roots near ericaceous shrubs is also interesting. Several studies have demonstrated that some fungi in this family could have a dual-mode of root colonization and forms ectomycorrhiza as well as ericoid mycorrhiza (Vrålstad et al., 2000; Villarreal-Ruiz et al., 2004; Grelet et al., 2010). These species could be more efficient to provide nutrients to ericaceous shrubs than to BF saplings and could explain the lower N nutrition of BF near ericaceous shrubs.

The lower nutrient concentrations found in fir needles (N and K) under BSE stands compared to TA stands (Figure 2), suggest a negative effect of black spruce and ericaceous shrubs association on BF seedlings. These results are consistent with previous studies where ericaceous shrubs that are associated with non-EMF fungi may compete with EMF-associated trees (Yamasaki et al., 1998; Walker et al., 1999; Mallik, 2003). Ericoid mycorrhizal fungi are particularly competitive for recalcitrant organic matter (ROM) decomposition and uptake of N and P (Read, 1996) and probably have the ability to take up nutrient from ROM found around ericaceous shrubs (Joanisse et al., 2018) contrarily to BF-associated EMF communities. In hardwood forests of the southern Appalachians, the presence of *Rhododendron maximum* L. reduced the ramification index of eastern hemlock (*Tsuga canadensis* [L.] Carrière) saplings four-fold (Walker et al., 1999). In our study, the presence of ericaceous shrubs (associated with ericoid fungi) close to fir saplings rather affected their root EMF communities than reduced the ramification index. Numerous studies have already shown how ericaceous shrubs affect EMF communities associated with trees, such as red oak (*Quercus rubra* L.), hemlock (Walker et al., 1999), pine (*Pinus strobus* L., *P. sylvestris* L.) (Kohout et al., 2011), and black spruce (Yamasaki et al., 1998; Kennedy et al., 2018). In the boreal forest, ericaceous shrubs not only compete with fir growth and nutrition, but also modify forest dynamics and lead to thick accumulation of soil organic matter and soil acidification. This phenomenon, is a recurrent problem within boreal forests that are situated in the Ontario-Quebec Clay Belt and is often associated with the presence of ericaceous shrubs and Sphagnum mosses (Fenton et al., 2005) and a loss of forest productivity (Simard et al., 2007). The thickness of the soil organic layer is correlated with a decrease in TA establishment and growth within the black spruce-feather moss domain (Lafleur et al., 2015), together with a decrease in black spruce establishment in sites that are dominated by *K. angustifolia* L. (Mallik and Kravchenko, 2018). Our results suggest that forest regeneration failure in areas with high ericaceous shrub abundance could be explained by their effect on EMF communities, and reciprocally, invite to consider below-ground interactions to avoid or limit regeneration failure.

Whilst our study focused on fir growth, we revealed stronger differences in foliar N concentrations and ramification indices between stands. Indeed difference in annual growth between stands was detected only in 2015. Annual growth of trees is relatively slow in the boreal forest and is correlated with the length of the growing season, which can differ from 1 year to the next (Jarvis and Linder, 2000). Differences in early growth could thus be difficult to detect over a short period of time and would be probably more pronounced when studying several years of growth (see Arbour and Bergeron, 2011). Measuring fitness is always difficult for young trees over a short period of study and measures of foliar nutrient concentrations were more useful to detect differences between stands, and reflected the benefits of EMF symbiosis. Foliar nutrient concentrations were generally greater under TA compared to BS and correlated with growth, which confirmed our hypothesis. This result could be linked to the greater availability of nutrients in TA stands than in BS stands (Cavard et al., 2011; Nagati et al., 2018).

A major goal of forest ecology today is to determine ecosystem trajectories in a context of climate change. In the case of the balsam fir-black spruce forest ecotone, it appears that trembling aspen stands would provide a favorable niche for fir establishment and growth. As demonstrated by Arbour and Bergeron (2011), the higher abundance of balsam fir in TA than BS stands is more pronounced for saplings than seedlings. This result translates a lower mortality and better growth of balsam fir in TA stands which leads to a greater abundance of mature and reproductive trees in these stands. This in turn may result in an increase of mixed forests and deep changes in ecosystem functioning. The distributional ranges of numerous tree species are likely to change within the context of climate change (Bellard et al., 2012; Iverson and McKenzie, 2013; IPCC Climate Change, 2014). Migration has already begun for many tree species in North America (Brandt, 2009; Woodall et al., 2009). Their geographic ranges have been extending northward rapidly (up to 100 km/century; Woodall et al., 2009). Our results suggest that the climatic niche could not alone explain species abilities to establish and that the mutualistic niche (sensus Peay, 2016) have to be explored to ensure a better comprehension of tree migration processes.

## Data Availability

All datasets for this study are included in the manuscript and/or the Supplementary Files.

## Author Contributions

MG, YB, MN, MR, and AD designed the study and interpreted the results. MN and MR carried out the field and laboratory work. MN and SM carried out the molecular biology and bioinformatics. MN carried out the statistical analyses. MN, MG, and MR wrote the manuscript. All authors edited and approved the final version of the manuscript.

## Conflict of Interest Statement

The authors declare that the research was conducted in the absence of any commercial or financial relationships that could be construed as a potential conflict of interest.

## Footnotes

1 ftp://ftp.ncbi.nih.gov/genbank/

2 https://unite.ut.ee/
